# Chemoinformatics-driven classification of Angiosperms using sulfur-containing compounds and machine learning algorithm

**DOI:** 10.1186/s13007-022-00951-6

**Published:** 2022-11-05

**Authors:** Muhammad-Redha Abdullah-Zawawi, Nisha Govender, Mohammad Bozlul Karim, Md. Altaf-Ul-Amin, Shigehiko Kanaya, Zeti-Azura Mohamed-Hussein

**Affiliations:** 1 Institute of Systems Biology (INBIOSIS), Universiti Kebangsaan Malaysia, 43600 UKM Bangi, Malaysia; 2 UKM Medical Molecular Biology Institute (UMBI), Jalan Yaacob Latif, Bandar Tun Razak, 56000 Cheras Kuala Lumpur, Malaysia; 3 Graduate School Information Science, Nara Institute of Science and Technology, 8916-5 Takayama-cho, Ikoma Nara 630-0192 Japan; 4 Department of Applied Physics, Faculty of Science and Technology, Universiti Kebangsaan Malaysia, 43600 UKM Bangi, Malaysia

**Keywords:** Angiosperms, Chemoinformatics, KNApSAck database, Sulfur-containing compounds, Molecular fingerprints, Monocot-dicot

## Abstract

**Background:**

Phytochemicals or secondary metabolites are low molecular weight organic compounds with little function in plant growth and development. Nevertheless, the metabolite diversity govern not only the phenetics of an organism but may also inform the evolutionary pattern and adaptation of green plants to the changing environment. Plant chemoinformatics analyzes the chemical system of natural products using computational tools and robust mathematical algorithms. It has been a powerful approach for species-level differentiation and is widely employed for species classifications and reinforcement of previous classifications.

**Results:**

This study attempts to classify Angiosperms using plant sulfur-containing compound (SCC) or sulphated compound information. The SCC dataset of 692 plant species were collected from the comprehensive species-metabolite relationship family (KNApSAck) database. The structural similarity score of metabolite pairs under all possible combinations (plant species-metabolite) were determined and metabolite pairs with a Tanimoto coefficient value > 0.85 were selected for clustering using machine learning algorithm. Metabolite clustering showed association between the similar structural metabolite clusters and metabolite content among the plant species. Phylogenetic tree construction of Angiosperms displayed three major clades, of which, clade 1 and clade 2 represented the eudicots only, and clade 3, a mixture of both eudicots and monocots. The SCC-based construction of Angiosperm phylogeny is a subset of the existing monocot-dicot classification. The majority of eudicots present in clade 1 and 2 were represented by glucosinolate compounds. These clades with SCC may have been a mixture of ancestral species whilst the combinatorial presence of monocot-dicot in clade 3 suggests sulphated-chemical structure diversification in the event of adaptation during evolutionary change.

**Conclusions:**

Sulphated chemoinformatics informs classification of Angiosperms via machine learning technique.

**Supplementary Information:**

The online version contains supplementary material available at 10.1186/s13007-022-00951-6.

## Background

Angiosperms or flowering plants bearing seeds represent the largest group of living plants. With up to 286000 different species found on land areas, they exist in various forms displaying a wide spectrum of differences in embryology, organ-specific anatomy, micromorphology, palynology and others [1]. Plants produce structurally unique compounds (secondary metabolites) such as the polyphenols, alkaloids, terpenes, phenolics, flavonoids and glucosinolates that may or may not significantly support functional roles such as basic processes in growth, development and physiology [2]. The chemical features of natural products are gaining complexity in terms of the content, composition, structure, cellular localization and distribution. The present-day classification of Angiosperms follows morphological characteristics for species-level distinction. There are two major groups in Angiosperms: (i) dicotyledons; seeds with two cotyledons, tap root and leaves with net-like venation and, (ii) monocotyledons; seeds with single cotyledons, adventitious root and leaves with parallel venation. Since plants are bestowed with a broad chemodiversity, these chemical information are harnessed as taxonomy markers in plant natural system classifications [3–5]. Nevertheless, no studies have attempted to classify higher taxa plants using chemical information solely as integrative methods are rendered much powerful.

Plant sulfur-containing compounds (SCCs) are S-containing amino acid-derived secondary metabolites [6–8]. S is the fourth most essential nutrient to plants after nitrogen, phosphorus and potassium. The S assimilation pathway serves as the precursor for SCC and associated metabolite biosynthesis; methionine, cysteine and phenylalanine amino acids, *S*-adenosylmethionine coenzyme and glutathione prosthetic groups [9]. In general, SCCs are involved in essential biological activities such as host induced defense responses against microbes and herbivores [10, 11], oxidative stress responses and mitigation of heavy-metal toxicity [12]. SCCs display broad chemodiversity which includes glucosinolates, phytosulfokines, sulphated flavonoids and sulfooxy derivatives [13]. In glucosinolates, also the largest group of SCCs, there are about 120 different forms described in higher plants [14]. The SCCs are distributed in numerous species, stretching from grass family (wheat, barley, oat), vegetables (tomato, broccoli, carrot, celery) and fruit trees [15, 16].

Chemotaxonomic studies for the classification of plant species have been conducted at various levels with different types of chemical compounds (taxonomic markers), mainly secondary metabolites. For example, species-level differentiation of *Hedysarum* genus was achieved using chemical profiles of isoflavonoids, chalcones, benzofurans, comestans and pterocarpenes [2], *Solanum torvum* was distinguishable from its closely related member, *S. erianthum* using the information from phenolic markers such as delphinidin 3,5-O-diglucoside and malvidin 3-O-arabinoside and 24-methyllathosterol ferulate. Within Selenastraceae, fatty acid methyl ester (FAME)-based-chemotaxonomy was successfully used to resolve the uncertainties encountered from using the molecular approach [17]. The significant explosion of metabolomics data and databases coupled with machine learning algorithms inform new knowledge in plant research [18–23].

In this study, the graph clustering algorithm (DPClusO) was applied for the identification of overlapping clusters with similar structural SCCs. The DPClusO algorithm generates high density clusters and has been adopted in big data analyses such as protein–protein interactions [24], identification of functional gene relations from gene expression datasets [25], pathway prediction [26] and many others [27]. Chemical information offers important insights into biochemical systematics, however, the scope of SCC chemical structure information to draw organizational concepts in flowering plants is underexplored. Presently, very few studies have attempted to use SCCs as taxonomy markers for plant system classification. Herein, chemoinformatics approach which integrates metabolite-content and structure similarity information of SCCs are applied for Angiosperms classification.

## Results

### SCC-producing Angiosperms: distribution and structural similarities

**Fig. 1:**
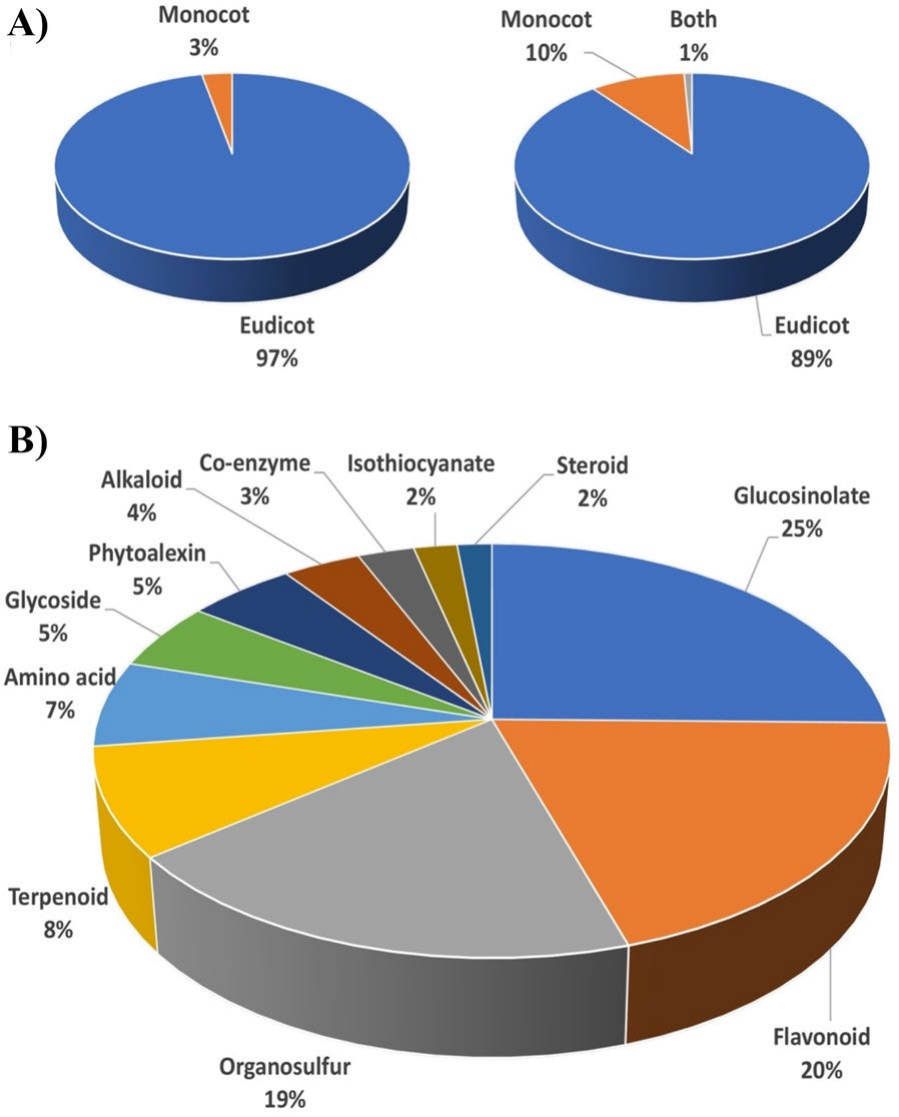
Sulfur-containing compounds (SCCs) in Angiosperms. **A** Distribution of monocots and eudicots with SCCs. **B** Distribution of monocots and eudicots with unique (orange and blue) and common (grey) SCCs. **C** Distribution of the different types of SCCs in Angiosperms. All values are generated from a total of 450 plant species and 491 SCCs, retrieved from the KNApSAcK database

A total of 2253 species-metabolite binary relations associated with 552 sulfur-containing compounds (SCCs) and 692 plant species were obtained from KNApSAcK Core DB. Of which, 450 species (with at least two SCCs) with 491 SCCs engaged in a total of 2011 species-metabolite relations were fed into the analysis. Figure 1 shows the distribution of SCCs in eudicots and monocots of Angiosperms. About 97% of the total plants were SCC-producing plants (436 eudicots) whilst the remaining small percentage were monocots (Fig. 1A). A total of 439 (89%) and 48 (10%) SCCs were uniquely present in eudicot and monocot, respectively. The following SCCs were common in both eudicots and monocots: dipropyl disulfide (C00001247), propane-1-tiol (C00001267), malonyl-CoA (C00007260) and 4-coumaroyl-CoA (C00007280) (Fig. 1B). The SCCs were annotated into 11 different classes described as following: flavonoid, steroid, iso-thiocyanate, co-enzyme, alkaloid, amino acid, terpenoid, glucosinolate, phytoalexin, organosulfur and allicin. Glucosinolate is the most abundant (25% of total SCCs) class (124 SCCs), followed by flavonoids (98 SCCs), organosulfur (94 SCCs), terpenoids (41 SCCs) and amino acids (33 SCCs). The iso-thiocyanate and steroid classes represent 2% of the total SCCs (Fig. 1C).

**Fig. 2:**
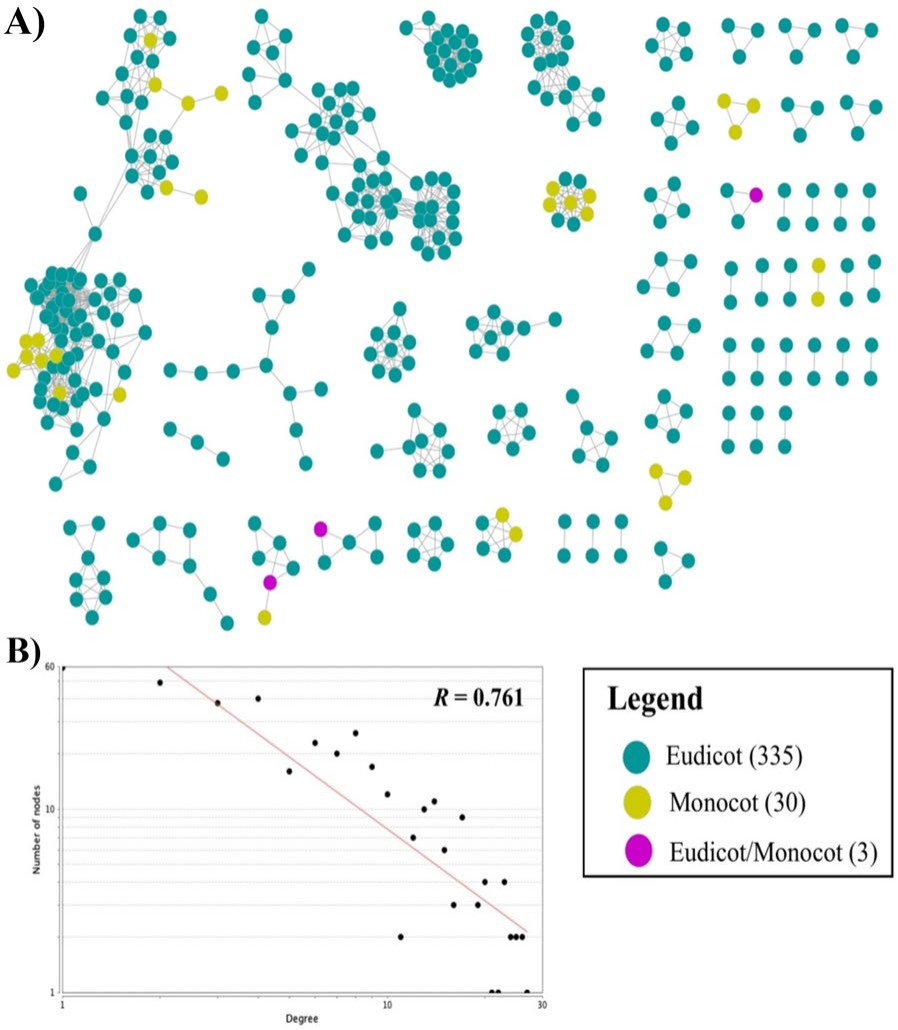
Structural similarity network of sulfur-containing compounds (SCCs). Nodes represent the SCCs and edges (grey lines) indicate correlation at Tanimoto coefficient > 0.85. Coloured nodes are represented as following: (i) yellow node; SCC of monocot, (ii) green node; SCC of eudicot and iii) purple node; SCC present in both eudicot and monocot. **A** Structural similarity- network visualized using Cytoscape ver. 3.7. **B** Network degree distributions in log-scale computed using NetworkAnalyzer

A total of 4783 metabolite pairs with a Tanimoto coefficient > 0.7 were obtained and 1,200 metabolite pairs with a Tanimoto coefficient > 0.85 were selected for network construction. The structural similarity network consists of 368 SCCs, with 105 single nodes (Fig. 2A). Single node denotes SCC with non-significant structural similarity score. A total of 335 and 30 SCCs were unique to eudicots and monocots, respectively and three SCCs were present in both eudicot and monocot plants. The degree of network distributions, as determined by power-law elucidated associations between two or more neighbouring nodes [40] (Fig. 2B). Only three SCCs common to eudicot and monocot (purple nodes) showed interactions within the sub-network (Fig. 2). Table 1 shows the metabolite pairs of similar structure SCCs in monocot and eudicot plants. The CoA-containing compounds were present in the following pairs: 1,2 (2-enoyl CoA) and 3 (Acyl CoA). Pair 4 were similar by amino acid grouping whilst pair 5 represented the −OH containing thiosulfinates (dihydroasparagusic acid, asparagusic acid, isobrugierol, brugierol and 3,4-epithiobutyl nitrile). Pair 6, volatile metabolites with an unpleasant odour are sulfide bond containing compounds (hydrogen sulfide, dimethyl disulfide, methyl mercaptan and methyl allyl disulfide). More than half of the metabolite pairs present in both monocot and eudicot plants (pair 7–16) were sulphated flavonoids, a rare representation of flavonoid derivatives (Table 1).

**Table 1:** Sulfur-containing compounds **(**SCCs) in monocot and eudicot plants and their pair-wise structural similarity at Tanimoto coefficient > 0.85

pair	KNApSAcK ID	SCC	Plant	KNApSAcK ID	SCC	Plant
1	C00007263	Cinnamoyl-CoA	M	C00007280	4-Coumaroyl-CoA	M/D
2	C00007280	4-Coumaroyl-CoA	M/D	C00007264	Feruloyl-CoA	D
				C00007281	Caffeoyl-CoA	D
3	C00007260	Malonyl CoA	M/D	C00007259	Acetyl-CoA	D
				C00007269	Acetoacetyl-CoA	D
4	C00001267	Propane-1-thiol	M/D	C00001379	l-Methionine	D
				C00001365	l-Homocysteine	D
5	C00000305	Dihydroasparagusic acid	M	C00048433	Isobrugierol	D
	C00000304	Asparagusic acid	M	C00007668	3,4-Epithiobutyl nitrile	D
				C00048339	Brugierol	D
6	C00001257	Methyl allyl disulfide	M	C00007266	Hydrogen sulfide	D
	C00001246	Dimethyl trisulfide	M	C00007323	d-Cysteine	D
	C00001266	Propanethial S-oxide	M	C00001351	l-Cysteine	D
	C00001245	Dimethyl disulfide	M	C00001258	Methyl mercaptan	D
	C00000747	S-Methyl cysteine	M			
7	C00004457	Tricetin 7,3ʹ-disulfate	M	C00004355	6-Hydroxyluteolin 7-sulfate	D
				C00004328	Luteolin 7-sulfate	D
				C00004428	8-Hydroxyluteolin 7-sulfate	D
8	C00004367	Luteolin 4ʹ-methyl ether 3ʹ-sulfate	M	C00004328	Luteolin 7-sulfate	D
9	C00004329	Luteolin 3ʹ-sulfate	M	C00004328	Luteolin 7-sulfate	D
				C00004958	Quercetin 3ʹ-O-sulfate	D
10	C00004331	Luteolin 7,3ʹ-disulfate	M	C00004406	6-Hydroxyluteolin 3ʹ-methyl ether 7-sulfate	D
				C00004328	Luteolin 7-sulfate	D
				C00004969	Isorhamnetin 7-O-sulfate	D
				C00004957	Quercetin 7-O-sulfate	D
11	C00004368	Luteolin 4ʹ-methyl ether 7,3ʹ-disulfate	M	C00004965	Quercetin 3,7,3ʹ,4ʹ-tetra-O-sulfate	D
				C00004412	6-Hydroxyluteolin 6,3ʹ-dimethyl ether 7,4ʹ-disulfate	D
				C00004411	6-Hydroxyluteolin 6,3ʹ-dimethyl ether 7-sulfate	D
				C00004328	Luteolin 7-sulfate	D
				C00004972	Isorhamnetin 3,7,4ʹ-tri-O-sulfate	D
12	C00004356	Luteolin 3ʹ-methyl ether 7-sulfate	M	C00004412	6-Hydroxyluteolin 6,3ʹ-dimethyl ether 7,4ʹ-disulfate	D
				C00004411	6-Hydroxyluteolin 6,3ʹ-dimethyl ether 7-sulfate	D
				C00004328	Luteolin 7-sulfate	D
				C00004969	Isorhamnetin 7-O-sulfate	D
13	C00011343	Malvidin 3-glucoside-5-(2ʺ-sulfatoglucoside)	M	C00006073	Tamarixetin 3-glucoside-7-sulfate	D
				C00013898	Quercetin 3-glucoside-3ʹ-sulfate	D
14	C00006086	Isoorientin 7-O-sulfate	M	C00006074	Patuletin 3-glucoside-7-sulfate	D
15	C00006087	Vitexin 7-O-sulfate	M	C00004500	Isoscutellarein 4ʹ-methyl ether 8-(2ʺ-sulfatoglucuronide)	D
				C00004498	8-Hydroxyapigenin 8-(2ʺ-sulfatoglucuronide)	D
				C00004253	Isoscutellarein 4ʹ-methyl ether 8-(2ʺ-sulfatoglucoside)	D
				C00013648	Isoscutellarein 4ʹ-methyl ether 8-(2ʺ,4ʺ-disulfatoglucuronide)	D
				C00013644	8-Hydroxyapigenin 8-(2'',4ʺ-disulfatoglucuronide)	D
16	C00006084	Orientin 7-O-sulfate	M	C00004500	Isoscutellarein 4ʹ-methyl ether 8-(2ʺ-sulfatoglucuronide)	D
				C00006075	Gossypetin 8-glucoside-3-sulfate	D
				C00004498	8-Hydroxyapigenin 8-(2ʺ-sulfatoglucuronide)	D
				C00045108	Theograndin II	D
				C00004427	8-Hydroxyluteolin 8-glucoside-3ʹ-sulfate	D
				C00004435	8-Hydroxyluteolin 4ʹ-methyl ether 8-glucoside-3ʹ-sulfate	D

*M* monocot, *D* eudicot, *M/D* monocot and dicot

### Association between metabolite similarity and biological function

**Fig. 3:**
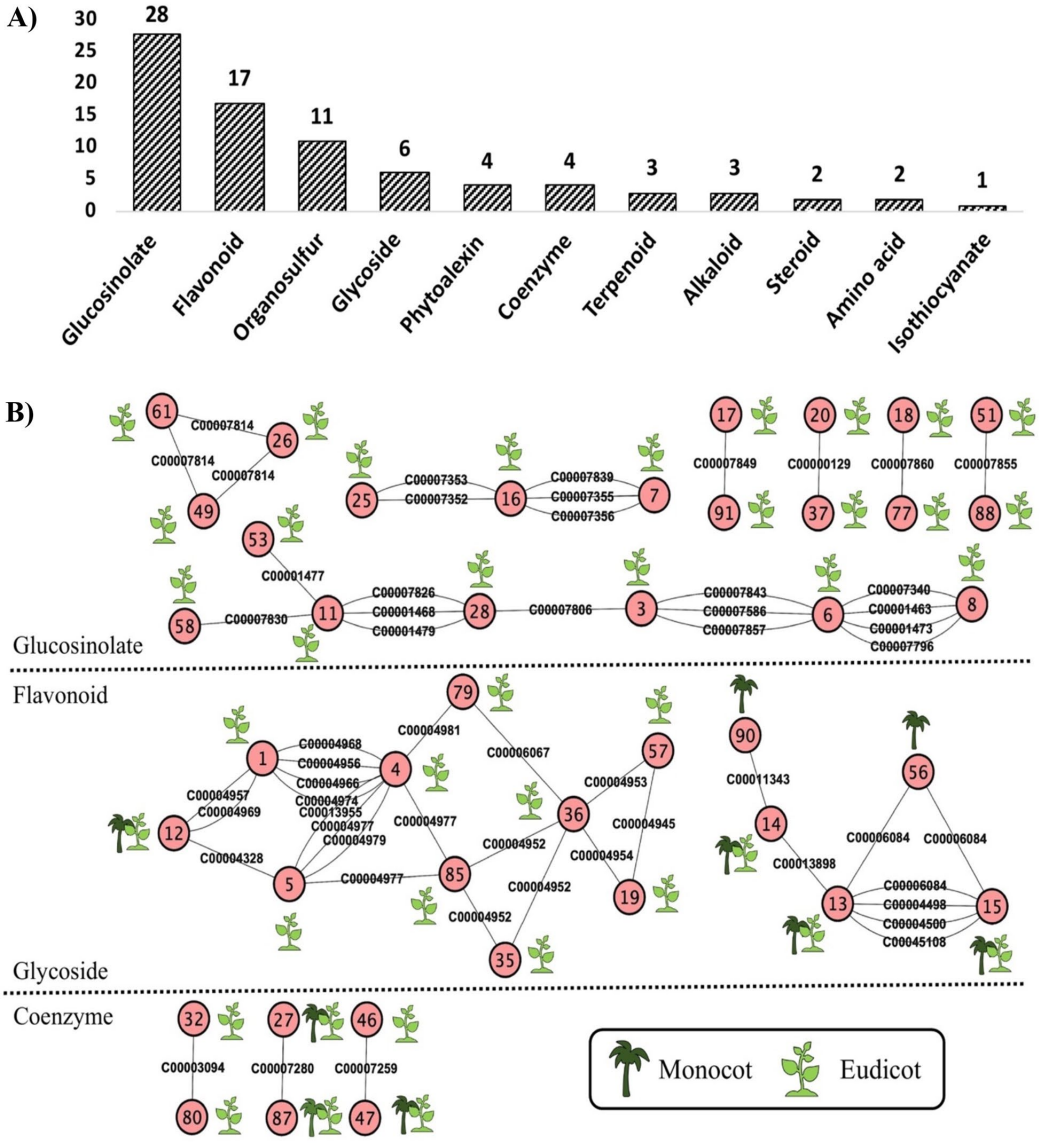
Structural similarity clustering by DPClusO algorithm. **A** Distribution of sulfur-containing compound (SCC) clusters. **B** Network of overlapping clusters obtained from the network clustering analysis. Grey line indicates SSC association between the clusters. Red node represents the KNApSAcK cluster ID and the SCCs are denoted as C000XXXXX-edges (grey line)

A total of 92 clusters were built with 356 different SCCs; 42 clusters showed association with two or more metabolites (overlapping). The clusters were grouped according to classes of SCCs; glucosinolate, flavonoid, organosulfur, glycoside, phytoalexin, coenzyme, terpenoid, alkaloid, steroid, amino acid and isothiocyanate. Clusters containing glucosinolates showed the highest distribution at 28, followed by clusters of flavonoids (17), organosulfur (11) and glycoside (6) compounds. Clusters with less than five SCCs were comprised of phytoalexin, coenzyme, terpenoid, alkaloid, steroid, amino acid and isothiocyanate compounds (Fig. 3A). Under the network presentation, the flavonoid containing overlapping clusters showed the most number of associations. There were two free networks, each with 10 and 5 overlapping clusters. The network chain with total number of clusters = 10 was mainly represented by monocots. In cluster 12, both the monocot and dicot species were present. The small network chain with 5 overlapping clusters showed three clusters with a mixture of eudicots and monocots and the remaining were represented by eudicots only. Cluster 4, also the hub cluster showed association with four different clusters (1, 5, 79 and 85) through 9 different flavonoids indicated as following: cluster 4–5; C00013955, C00004977, C00084979, cluster 1–4; C0004968, C0004956, C0004966, C0004974, cluster79-4; C0004981 and cluster 4–85; C0004977 (Fig. 3B). In the glucosinolate containing overlapping cluster network, only dicot species were identified in all the individual clusters. There were only one big (> 3 clusters) network chain and 3 small chains (≤ 3 clusters). The biggest chain contained 7 individual clusters connected by 13 different glucosinolates. Two chains of three overlapping clusters were connected by 3 and 5 different glucosinolates. There are 4 independent pair-wise clusters connected by a single glucosinolate. Cluster 6 showed the highest number of interactions and appeared as the hub cluster in the glucosinolate overlapping cluster network. Cluster 3–6 were connected by C00007843, C00007586 and C00007857 while cluster 8–6 were connected by C00007340, C00001463, C00001473 and C00007796 (Fig. 3B). In the glycoside and coenzyme network of overlapping clusters, small pairwise networks were observed. The glycoside network of overlapping clusters was represented by monocots only whereas the coenzyme network of overlapping clusters showed a representation of both the eudicots in clusters 27–87 only. In clusters 46–47, monocots were present in cluster 46 only whilst cluster 47 showed a combination of monocots and dicots (Fig. 3B).

### Pathway enrichment and Angiosperm phylogeny

**Fig. 4:**
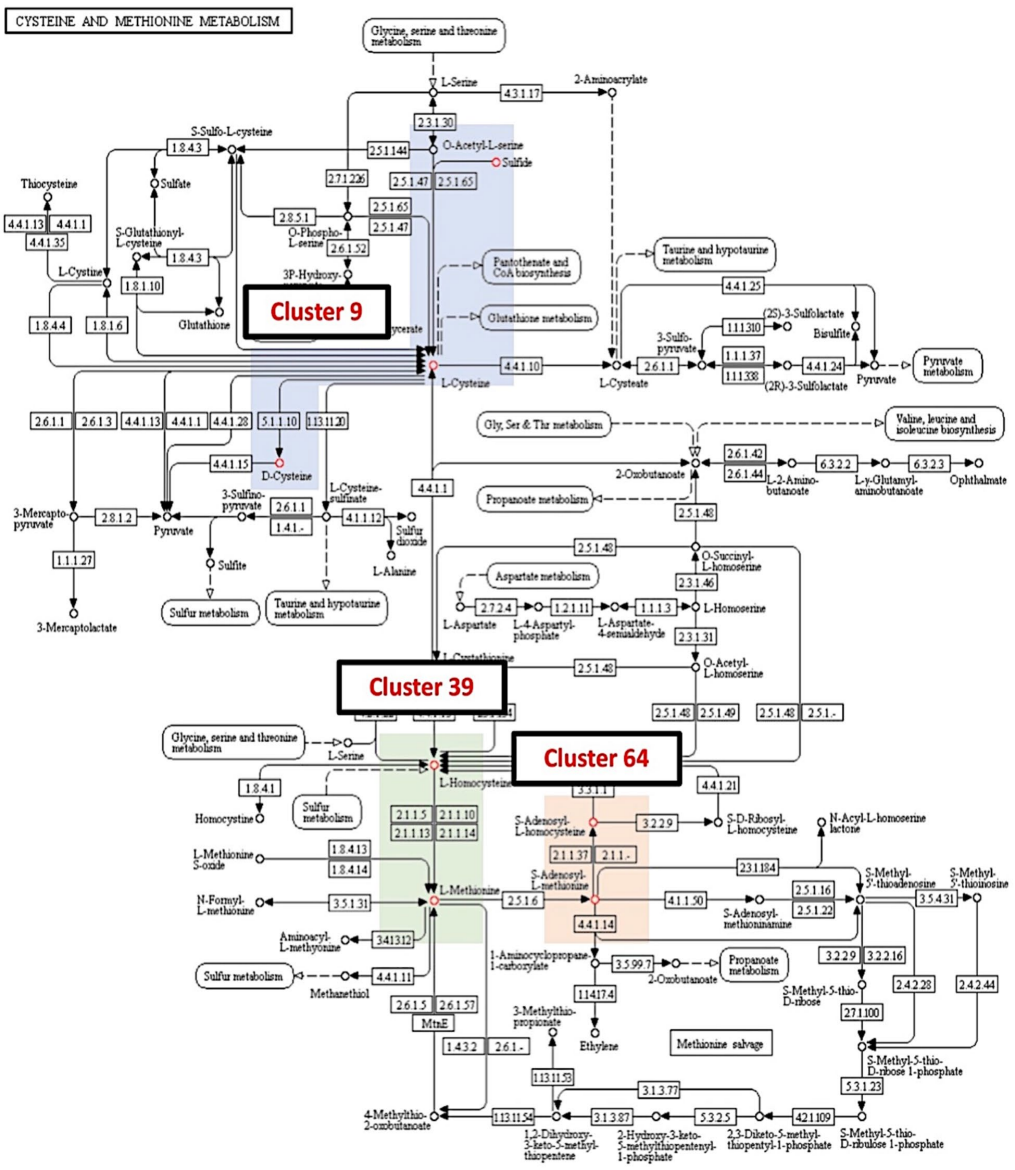
Metabolite clusters mapped within the cysteine and methionine metabolism (map00270) pathway. Coloured blocks represent clusters of unique sulfur-containing compounds (SCCs): blue; cluster 9, green; cluster 39 and orange; cluster 64

**Fig. 5:**
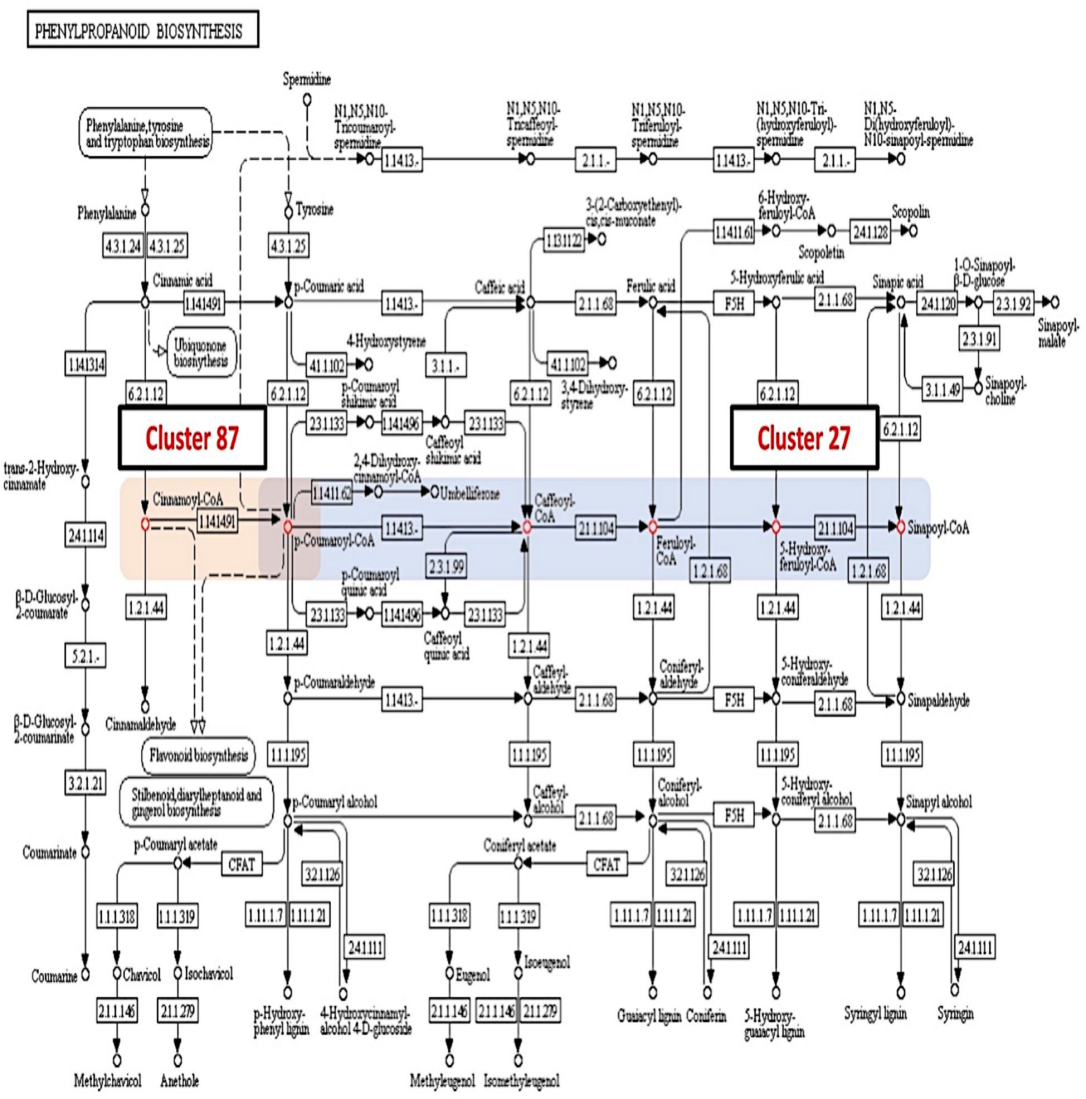
Metabolite clusters mapped within the phenylpropanoid biosynthesis (map00940) pathway. Coloured blocks represent clusters of unique sulfur-containing compounds (SCCs): blue; cluster 27, and orange; cluster 87

From a total of 356 SCCs, only 47 metabolites from 24 clusters were mapped into 53 KEGG metabolic pathways. A total of 23 clusters were involved in the secondary biosynthesis pathway (map01110) whilst 17 clusters showed participation in the 2-oxocarboxylic acid metabolism (map01210) and glucosinolate biosynthesis (map00966). Six clusters were involved in plant secondary metabolite biosynthesis (map01060), and four clusters in cysteine and methionine metabolism (map00270), phenylalanine metabolism (map00360), tryptophan metabolism (map00380), phenylpropanoid biosynthesis (map01061) and plant hormone (map01070) pathways. The SCCs in cluster 1 were involved in flavon and flavonol biosynthesis. The pathway-oriented clustering analysis showed that 23% of SCCs from a similar cluster were mapped within a similar pathway. For example, overlapping clusters composed of clusters 46 and 47 showed the presence of functionally related acetyl-CoA, malonyl-CoA and acetoacetyl-CoA intermediates in lipid, carbohydrate, and amino acid metabolism pathways. The cysteine and methionine metabolism pathway contained clusters 9, 39 and 64 (Fig. 4). In the phenylpropanoid pathway, both clusters 87 and 27 occupied a localized region within the pathway map (Fig. 5).

The pathway-oriented cluster mapping indicated that structurally similar metabolite clusters show localization in reaction steps within the KEGG pathway. Most of the metabolite cluster present in either the intermediary metabolism or specific metabolism of KEGG metabolic pathway maps. In the cysteine and methionine pathway map (map00270), the correlation between cluster 9, cluster 39, and cluster 64 governed the continuous reaction steps. Coumaroyl-CoA found present in cluster 27 and cluster 87 indicate an intermediatory role in the propanoid biosynthesis. In the cysteine and methionine metabolism pathway, three metabolites from cluster 9 and two metabolites each from clusters 39 and 64 were found through the pathway-oriented cluster mapping (Fig. 4). The pathway map is divided into two regions of cysteine pathway (cluster 9) and methionine pathway (cluster 39 and cluster 64). For overlapping clusters, the localized regions of the pathways are highly intercepted between two or more metabolite clusters (Fig. 5). For example, two localized regions of cluster 27 and cluster 87 were intercepted at coumaroyl-CoA, a structurally similar metabolite present in both clusters.

The transformed species-SCC binary relations of 450 species and 491 SCCs produced a 450 × 227 binary matrix. The 450 plant species were classified into 50 hierarchical clusters and each cluster represented plants with a similar class of SCC content. The Angiosperms phylogeny with three distinct clades suggests that plant species with similar metabolite content were much closely related within the hierarchical cluster. Clade 1 and 2 were represented by eudicots only (total plants, 244) whilst clade 3 contained a mixture of both eudicot and monocot plants. The hierarchical cluster delineated Angiosperms into clade 1 and clade 2 of 244 eudicots and clade 3, a mixture of 14 monocots and 192 eudicots. A detailed view of the Angiosperm phylogeny species and pathway description are provided in Additional files 1 and 2.

**Fig. 6:**
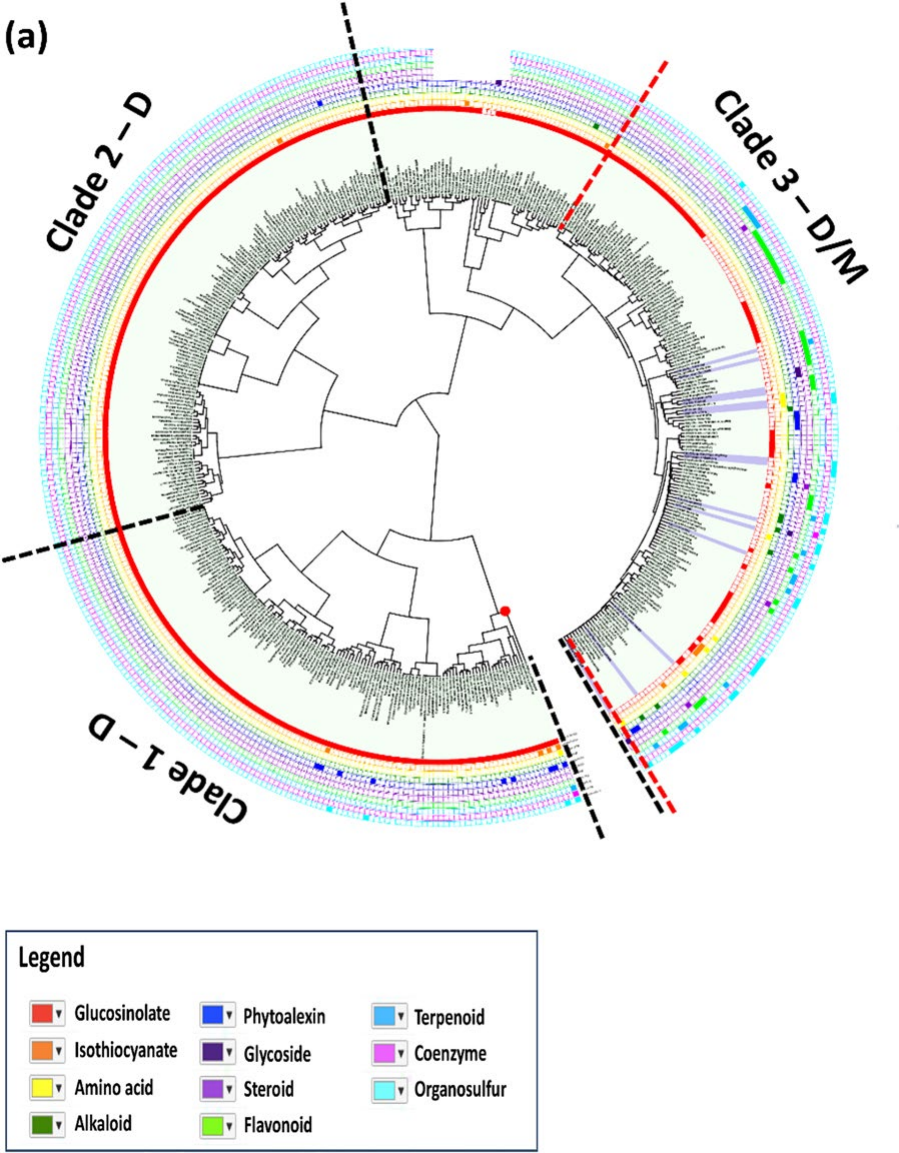
Hierarchical clustering of Angiosperms based on the sulfur-containing compound (SCC) content. A total of 50 metabolite clusters are divided into three main clades; Clade 1-D, Clade 2-D and Clade 3-D/M. Red dotted line denotes the position of *A. thaliana* in the phylogenetic tree. Clades are represented along the type of plant species indicated as following: *D* eudicot, *M* monocot, and *D/M *eudicot and monocot. The enlarged version of each clade is presented in S Fig. 7

At an average hierarchical clustering value of 50, a total of 46 plant species (92%) represented the eudicot clusters, one (2%) corresponded for monocot cluster and three in monocot-dicot, in combination cluster. Generally, 80% (40) of the clusters, were comprised of eudicots mainly. Among them, 70% of the eudicots were glucosinolate producers. The remaining eudicots from cluster 1, cluster 8, cluster 10, and cluster 44 found in clade 3 showed production of various forms of SCCs (Fig. 6). Similar clusters comprised of both monocot and eudicot plants were found in cluster 1, cluster 5 and cluster 43. In cluster 1, two monocots (*Zingiber officinale* and *Asparagus officinalis*) and a single eudicot (*Bruguiera gymnorhiza*) produced dithiolan and sulfonic acid. Meanwhile, dipropyl disulfide present in *Allium *sp*.* (monocot) and *Petiveria alliacea* (eudicot) were in similar sub-clade under cluster 1. Most of the metabolites from cluster 5 and cluster 43 are composed of SCC from the flavonoid class (Additional file 2). For instance, flavonol O-glycoside, a sulphated flavonoid was highly distributed in eudicot, whereas the flavone C-glycoside or glycoflavone was observed in monocot. Glycoflavone, such as vitalexin, orientin and luteolin were more abundant in monocots as compared to the eudicots [28–32].

## Discussion

The sample number poses biasness to a certain extent (eudicot number > monocot number), however, the network clustering performed using DPClusO algorithm corrected for the sampling bias error [33]. Based on Angiosperms chemo-information available in the KNApSAck database, a total of 450 different plant species with SCCs was identified. The Angiosperms selected for classification were represented by eudicots, mostly (97% of the total plants). The SCC distribution among the eudicots was much greater as compared to the monocots. Glucosinolate was ranked as the most abundant class of SCC in Angiosperms whereas the isothiocyanate and steroid emerged as the smallest class. From the ecological point of view, glucosinolates are rendered as natural pesticides, posing toxicity to a wide range of organisms from insects, bacteria, fungi, nematodes and mammals [34]. Glucosinolates inherent a chemically stable structure and remain biologically inactive within the sub-cellular compartments distributed within the plant tissues. Glucosinolates are activated by physical actions such as tissue damage, chewing and food processing. The glucosinolate-myrosinase system functions as plant natural defense system against insects and specialized receptor cells stimulated by defoliating pests (eggs and larvae). Upon physical injury, the endogenous enzyme myrosinase hydrolyzes glucosinolate into toxic and antinutritional biologically active products [14]. The glucosinolate representation is inversely proportional to its breakdown products which includes isothiocyanates, oxzzolidine-2-thiones, nitriles, epithionitriles, organic cyanides, oxazolidinethiones and ionic thiocyanate. This explains the association between glucosinolate and the degraded by-products in plant defense system. Flavonoids and organosulfur represented the second most abundant class with up to 98 and 94 SCCs, respectively. With over 5000 chemical structures, the flavonoids display broad diversity and hence, a broad range of functional roles in relation to plant’s survival. They impart important roles in numerous plant physiology and ecology-related processes such as seed and flower petal coloration, pollen germination, regulator of plant growth and protection against biotic and abiotic stressors.

Metabolites with high similarity scores are likely to be involved in similar biological functions [34, 35]. In general, the overlapping clusters obtained in this study displayed a similar metabolite function [36]. Glucosinolate and sulphated flavonoid were distributed in most clusters. In cluster 2, prototribestin (steroid saponin) showed structural similarity with terpenoid-type saponins such as sandrosaponin, tribestin and zygophyloside [49]. In cluster 23, tryptophan derivative compound (3-indolylmethylthiohydroximate) was clustered with indole phytoalexin compounds. Likewise, indole phytoalexin compounds such as cyclobrassinin and indole glucosinolate, sinalbin A and sinalbin B were structurally similar. Metabolites in cluster 29, cluster 30 and cluster 40 were composed of SCCs derived from reaction steps involved in glucosinolate biosynthesis. As such, hexa-, penta- and tetra- homomethionine are Met derivatives involved in the initial step of glucosinolate side-chain elongation while isothiocyanate is the product of the glucosinolate degradation (Table 2).

**Table 2:** Description of organosulfur overlapping clusters comprised of structurally similar sulfur-containing compounds (SCCs)

Clusters	Class	SCCs	Description
2	Terpenoid (T), Steroid (S)	Prototribestin (S), Rotundioside A, B, J, K (T), Bacopaside I, III (T), Zygophyloside O, P (T), Tribestin (T), Sandrosaponin II, III, V, VI, VIII (T), Bacopaside VI (T)	Known as saponin compounds
9	Amino acid (AA), Organosulfur (O)	L-S-methylcysteine (AA), L-Cysteine (AA), D-Cysteine (AA), Dimethyl di-, tri-sulfide (O), Methyl allyl disulfide (O), Methyl mercaptan (O), Propanethial S-oxide (O), Hydrogen sulfide (O)	Metabolite from continuous sets of reaction steps in alliin metabolism
23	Phytoalexin (P), Organosulfur (O)	3-Indolylmethylthiohydroximate (O), Methoxybrassinin (P), Brassinin (P), Brassitin (P), Sinalexin (P), Brassicanal A, B, C (P), Brassilexin (P)	Known as indole compounds
29	Organosulfur (O), Isothiocyanate (I), Amino acid (AA)	Hexahomomethionine (AA), (R)-8-Methylsulfinyloctyl isothiocyanate (I), 2-Oxo-10-methylthiodecaonoic acid (O), 9-Methylthiononanaldoxime (O)	Metabolite from continuous sets of reaction steps in glucosinolate biosynthesis
30	Organosulfur (O), Isothiocyanate (I), Amino acid (AA)	Pentahomomethionine (AA), (R)-7-Methylsulfinyl heptyl isothiocyanate (I), 2-Oxo-9-methylthiononanoic acid (O), 8-Methylthiooctanaldoxime (O)	Metabolite from continuous sets of reaction steps in glucosinolate biosynthesis
33	Alkaloid (A), Organosulfur (O), Amino acid (AA)	Aglatenol (A), Dihomomethionine (AA), 2-Oxo-6-methylthiohexanoic acid (O), 5-Methylsufinylpentyl nitrile (O)	Unknown. Related according to a similar structure
38	Amino acid (AA), Organosulfur (O)	Alliin (AA), Asparagusic acid syn-S-oxide (O), Asparagusic acid anti-S-oxide (O)	Known as thiosulfinate compounds
39	Amino acid (AA), Organosulfur (O)	L-Methionine (AA), Propane-1-thiol (O), Homocysteine (AA)	Probably involved in glutathione-mediated detoxification I and II pathways
40	Amino acid (AA), Organosulfur (O)	Tetrahomomethionine (AA), 6-Methylthiohexanaldoxime (O), 2-Oxo-8-methylthiooctanoic acid (O)	Metabolite from continuous sets of reaction steps in glucosinolate biosynthesis
44	Phytoalexin (P), Glucosinolate (G)	Sinalbin A, B (G), Cyclobrassinin (P)	Known as indole compounds
92	Alkaloid (A), Phytoalexin (P)	Dithyreanitrile (A), Rapalexin A (P)	The final product of indole compound degradation

Structural similarities between the following sulphated flavonoids were observed in cluster 13, cluster 14 and cluster 15: malvidin 3-glucoside-5-(2ʺ-sulfatoglucoside) (C00011343) and orientin 7-O-sulfate (C00006084). Sulphated flavonoids unique to eudicot and monocot were structurally similar (cluster: 12, 13, 14, and 15). In Angiosperms, flavonoids are the most ubiquitously present natural products. Flavonoid sulfation is a conjugation reaction that utilizes sulfate group as donor and flavones, flavonols or their corresponding methyl esters as the acceptor molecules. The sulphated flavonoids are involved in reactive hydroxyl group detoxification, which directly contributes to the hydrophilicity of cellular compartment (solubility). Plants thriving in stressful environment assume sulfate ion sequestration for ecological adaptation. The sulphated flavonoids are naturally present in about 300 plant species comprised of eudicots and monocots [37, 38]. The findings corroborated with the present knowledge whereby nearing 50% representation of eudicot-monocot co-occurring metabolite pairs were all sulphated flavonoids. The distribution of SCCs were higher in the eudicots compared to monocots, and so does the structural diversity. The flavonoid containing overlapping clusters showed the most number of associations in the network, implying its broad spectrum functional roles. The interaction between the eudicot and monocot sulphated flavonoids suggests structural similarities and/or probable polyphyletic origin among the plant species. Each cluster represents a distinct entity of highly connected structural similarity and thus, may have been involved in similar biological functions [39].

Large clusters are often associated with a broad range of biological functions, in contrast to small clusters that have narrow and specific functions [24, 25]. In this study, the pathway-oriented cluster mapping displayed associations between the chemical composition and biochemical pathway. Glucosinolate was uniformly distributed among the Clade 1 eudicots. They were mainly members of the cabbage and mustard family (Brassicaceae), and others listed as the following: Erysimum (highest occurrence), Brassica, Lepidium, Cakile, Thelypodium, Wasabi, Alyssum, Cheiranthus, Malcomia, Eruca, Leavenworthia, Conringia, Iberis, Isatis, DiplotaxisLesquerella, Cardamine and Arabidopsis. Others such as the Gynandropsis from clade 1 represented a higher taxa of the Brassicales order. Members of clade 2 were similar to clade 1 in terms of consistent containment of glucosinolate compound. However, the eudicot members were comprised of several different families listed as the following genera: (i) Brassicaceae; Boechera, Arabis, Lunaria, Christoleaone, Sisymbrium, Thelypodium, Brassica, Crambe, Coincya, Descurainia, Fibigia, Nasturtiopsis, Matthiola, Capsella, Draba, Coincya, Selenia, Peltaria, Rorippa, Raphanus, Schouwia, Diplotaxis, Moricandia, Zilla, Cardamine and others (ii) Tropaeolaceae; Tropaeolum, (iii) Moringaceae; Moringa, (iv) Capparaceae; Capparis, Cleome (uniformly distributed under a single sub-clade), and v) Gyrostemonaceae; Tersonia.

## Conclusions

In this study, the chemoinformatics-driven phylogeny of Angiosperms showed parallel results with the traditional morphology-based classification to a great extent. Clade 1 and clade 2 of eudicots were distantly related to clade 3 of eudicot-monocot in combination. Glucosinolate compound was distributed among the species in clade 1–2. Amongst the different classes of SCCs, glucosinolate was ranked as the most abundant class whereas the isothiocyanate and steroid emerged as the smallest class. The flavonoids emerged as the second most abundant class after glucosinolate. Both glucosinolate and flavonoids have shown apparent structural diversity implicated in the trajectory of plant evolution driving the species chemo-diversity. The first is important in plant defense response, adaptability, tolerance against stressors and cellular level physiobiochemical activities, whereas, the latter plays a fundamental role in growth and development, and physiological processes.

## Methods

### Data collection and pre-processing

**Fig. 7:**
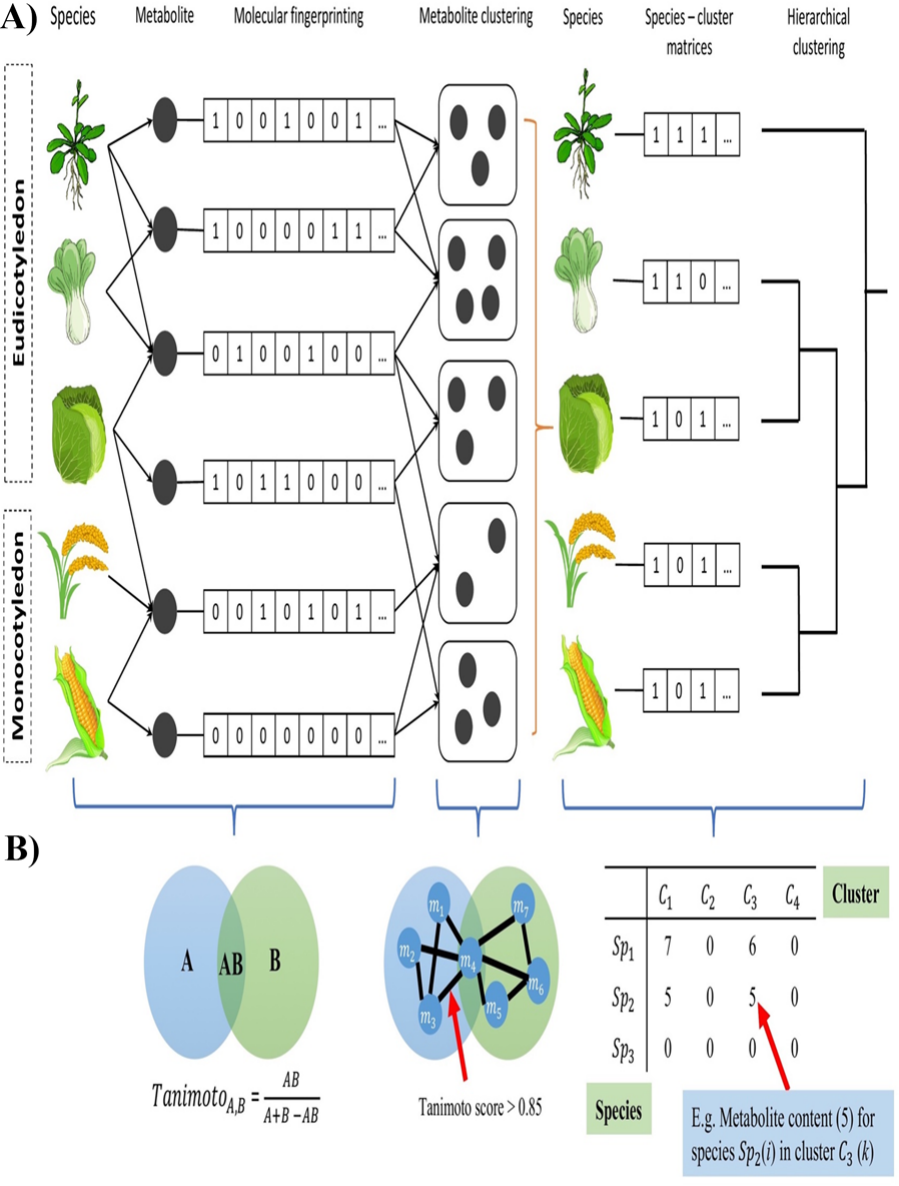
Schematic workflow for Angiosperms classification using sulfur containing compound (SCC) dataset. **A** The workflow is divided into three stages; (i) data collection and molecular fingerprinting of structural similarity based on Tanimoto score, (ii) clustering of metabolite pairs with Tanimoto score > 0.85, and (iii) hierarchical clustering. **B** Mathematical models supporting each stage described in (**A**)

Plant-specific sulfur-containing compounds (SCCs) were collected from KNApSAck Core DB and KNApSAck DB (https://www.knapsackfamily.com/knapsack). A total of 552 SCCs were identified from 692 plant species. Plants with less than two different SCCs were manually filtered out from the dataset. The corresponding.MOL files for all the identified metabolites were retrieved from the KNApSAcK Core DB. The SCCs were annotated via bibliomic search using the following databases: PubChem (https://www.pubchem.ncbi.nlm.nih.gov) [40], KEGG (https://www.genome.jp/) [41, 42] and Metlin (https://www.scripps.edu) [43]. Figure 7 illustrates the schematic workflow of the method employed in this study.

### Structural similarity analysis

The structural similarities of the identified SCCs were determined using the ChemmineR, an R-package, version 2.30.2 [44]. The atom pair fingerprints of all SCCs were generated from the.MOL metabolite structure input files and the structural similarities between pairs of metabolites were determined by Tanimoto coefficient. The Tanimoto coefficient values range from 0–1 (with increased value, the stronger the structural similarity), whereby 0 denotes no structural similarity and 1 indicates the highest similarity. The Tanimoto coefficient cut-off value was set at > 0.85 [45, 46]. All metabolite pairs were screened by Tanimoto coefficient and pairs that did not meet the cut-off value were filtered out from the metabolic network construction input data [47]. The metabolite network was visualized using Cytoscape software, version 3.6.1 [48].

### Sulfur-containing compound (SCC) cluster

DPClusO graph clustering algorithm was used for the identification of overlapping clusters from the metabolite network comprised of structurally similar SCCs pairs [49]. The parameters deployed in the algorithm for the cluster *k* are defined as follows: (i) cluster property (${{\varvec{c}}{\varvec{p}}}_{{\varvec{n}}{\varvec{k}}}$), (ii) density (${{\varvec{d}}}_{{\varvec{k}}}$), (iii) ratio of the edges $(\left|{{\varvec{E}}}_{{\varvec{k}}}\right|)$ and, (iv) maximum possible number of edges$(\left|{{\varvec{E}}}_{{\varvec{k}}}\right|{\varvec{m}}{\varvec{a}}{\varvec{x}})$. The ${{\varvec{d}}}_{{\varvec{k}}}$ was calculated using $\left|{{\varvec{E}}}_{{\varvec{k}}}\right|)$ and $\left|{{\varvec{E}}}_{{\varvec{k}}}\right|{\varvec{m}}{\varvec{a}}{\varvec{x}}$. ${{\varvec{N}}}_{{\varvec{k}}}$ represents the number of nodes in cluster *k*. The ${{\varvec{E}}}_{{\varvec{n}}{\varvec{k}}}$ denotes the total number of edges between the node, *n* and the cluster nodes [40]. The cluster property(${{\varvec{c}}{\varvec{p}}}_{{\varvec{n}}{\varvec{k}}}$) of node (*n*) in cluster *k* is shown below:

$${cp}_{nk}=\frac{|{E}_{nk}|}{{d}_{k} x |{N}_{k}|}$$

The overlapping mode was set with the following cluster property: ${cp}_{nk}$ = 0.5, ${d}_{k}$= 0.7, and minimum cluster size = 2.

### Metabolite content-based hierarchical clustering

The correlation values between the species metabolite content and groups of similar structure metabolites (SCCs) were stored in a matrix. Matrix (*M*) consists of two conditions: M_*ik*_; *k* groups of similar structure metabolites and *i* number of species, and M_*jk*_; *k* groups of similar structure metabolites and *j* number of species. The Euclidean distances (*d*) calculated between two different species (*i* and *j*) with *n* number of SCC clusters were fed into hierarchical clustering to infer the chemo-relationship among the species. When M_*ik*_ = 1, the species *i* contains at least one pair of metabolites with similar structures from group *k*, whereas M_*jk*_ = 0 denotes an absence of a metabolite cluster in species *j*. The distance formula is expressed below:

$$d\left(i,j\right)= \sqrt{{\sum }_{k=1}^{n}{({M}_{ik}-{M}_{jk})}^{2}}$$

The analysis was conducted using the *hclust* function from ChemmineR tool, an R library [44] and hierarchical clusters were visualized using the iTOL web server (https://www.itol.embl.de) [40].

### Pathway mapping

All SCCs identified in this study were converted to KEGG Ligand identifiers using the Hyperlink Management System and ID converter System (http://biodb.jp/) [50, 51]. Following conversion, the SCCs were mapped onto KEGG metabolic pathway using the KEGG Ligand database (https://www.genome.jp/kegg/ligand.html) [41, 42].

## Supplementary Information

**Additional file 1. Description of sulfur-containing compounds (SCCs) in Angiosperms.**

**Additional file 2. Descriptive annotation of sulfur-containing compound (SCC) clusters.**

## Data Availability

The chemoinfomatics data sets utilized in this study are available at https://www.knapsackfamily.com/knapsack.

## References

[1] Thorne RF (2000). The classification and geography of the flowering plants: dicotyledons of the class Angiospermae (subclasses Magnoliidae, Ranunculidae, Caryophyllidae, Dilleniidae, Rosidae, Asteridae, and Lamiidae). Bot Rev.

[2] Liu Y, Yang H, Liu Y, Wang W, Zhao Y, Chen H (2019). Chemotaxonomy studies on the genus *Hedysarum*. Biochem Syst.

[3] Wink M (2003). Evolution of secondary metabolites from an ecological and molecular phylogenetic perspective. Phytochemistry.

[4] Wink M, Botschen F, Gosmann C, Schäfer H, Waterman PG (2010). Chemotaxonomy seen from a phylogenetic perspective and evolution of secondary metabolism. Annu Plant Rev Online.

[5] Martucci MEP, De Vos RCH, Carollo CA, Gobbo-Neto L (2014). Metabolomics as a potential chemotaxonomical tool: application in the genus *Vernonia*
*Schreb*. PLoS ONE.

[6] Iranshahi V (2012). A review of volatile sulfur-containing compounds from terrestrial plants: biosynthesis, distribution and analytical methods. J Essent Oil Res.

[7] Kopriva S, Calderwood A, Weckopp SC, Koprivova A (2015). Plant sulfur and big data. Plant Sci.

[8] Bell L, Oloyede OO, Lignou S, Wagstaff C, Methven L (2018). Taste and flavor perceptions of glucosinolates, isothiocyanates, and related compounds. Mol Nutr Food Res.

[9] Wittstock U, Kliebenstein DJ, Lambrix V, Reichelt M, Gershenzon J, Romeo JT (2003). Glucosinolate hydrolysis and its impact on generalist and specialist insect herbivores. Integrative phytochemistry: from ethnobotany to molecular ecology. Recent advances in phytochemistry.

[10] Bednarek P (2012). Sulfur-containing secondary metabolites from *Arabidopsis thaliana* and other Brassicaceae with function in plant immunity. ChemBioChem.

[11] Piasecka A, Jedrzejczak-Rey N, Bednarek P (2015). Secondary metabolites in plant innate immunity: conserved function of divergent chemicals. New Phytol.

[12] Ravilious GE, Jez JM (2012). Structural biology of plant sulfur metabolism: from assimilation to biosynthesis. Nat Prod Rep.

[13] Gläser K, Kanawati B, Kubo T (2014). Exploring the *Arabidopsis* sulfur metabolome. Plant J.

[14] Fahey JW, Zalcmann AT, Talalay P (2001). The chemical diversity and distribution of glucosinolates and isothiocyanates among plants. Phytochemistry.

[15] Supiko K, Kosinova A, Vavrusa M (2022). Sulfated phenolic acids in plants. Planta.

[16] Hawkesford H, Marschner P (2012). Functions of macronutrients. Marschner’s mineral nutrition of higher plants.

[17] Mori CC, Bagatini IL, Garcia T, Parrish C, Vieira AAH (2018). Use of fatty acids in the chemotaxonomy of the family *Selenastraceae* (Sphaeropleales, Chlorophyceae). Phytochemistry.

[18] Altaf-Ul-Amin Md, Wada M, Kanaya S (2012). Partitioning a PPI network into overlapping modules constrained by high-density and periphery tracking. ISRN Biomath.

[19] Afendi FM, Okada T, Yamazaki M, Morita A, Nakamura Y, Nakamura K, Ikeda S, Takahashi H, Altaf-Ul-Amin M, Darusman LK, Saito K, Kanaya S (2012). KNApSAcK family databases: integrated metabolite-plant species databases for multifaceted plant research. Plant Cell Physiol.

[20] Abdullah AA, Altaf-Ul-Amin Md, Ono N, Sato T, Sugiura T, Morita AH, Katsuragi T, Muto A, Nishioka T, Kanaya S (2015). Development and mining of a volatile organic compound database. Biomed Res Int.

[21] van Santen JA, Jacob G, Singh AL (2019). The natural products atlas: an open access knowledge base for microbial natural products discovery. ACS Cent Sci.

[22] Kanaya S, Altaf-Ul-Amin Md, Aki MH, Huang M, Ono N, Ben HW, Begley TP (2020). Databases for natural product research. Comprehensive natural Products III.

[23] Capecchi A, Reymond JL (2021). Classifying natural products from plants fungi or bacteria using the COCONUT database and machine learning. J Cheminform.

[24] Altaf-Ul-Amin M, Tsuji H, Kurokawa K, Asahi H, Shinbo Y, Kanaya S (2006). DPClus: a density-periphery based graph clustering software mainly focused on detection of protein complexes in interaction networks. J Comput Aided Chem.

[25] Altaf-Ul-Amin M, Afendi FM, Kiboi SK, Kanaya S (2014). Systems biology in the context of big data and networks. Biomed Res Int.

[26] Altaf-Ul-Amin M, Shinbo Y, Mihara K, Kurokawa K, Kanaya S (2006). Development and implementation of an algorithm for detection of protein complexes 470 in large interaction networks. BMC Bioinformatics.

[27] Altaf-Ul-Amin M, Hirose K, Nani JV, Porta LC, Tasic L, Hossain SF, Kanaya S (2021). A system biology approach based on metabolic biomarkers and 474 protein–protein interactions for identifying pathways underlying schizophrenia and 475 bipolar disorder. Sci Rep.

[28] Brazier-Hicks M, Evans KM, Gershater MC, Puschmann H, Steel PG, Edwards R (2009). The C-glycosylation of flavonoids in cereals. J Biol Chem.

[29] Saito K, Yonekura-Sakakibara K, Nakabayashi R, Higashi Y, Yamazaki M, Tohge T, Ferni AR (2013). The flavonoid biosynthetic pathway in *Arabidopsis*: structural and genetic diversity. Plant Physiol Biochem.

[30] Liu K, Abdullah AA, Huang M, Nishioka T, Altaf-Il-Amin M, Kanaya S (2017). Novel approach to classify plants based on metabolite-content similarity. Biomed Res Int.

[31] Tohge T, De Souza LP, Fernie AR (2017). Current understanding of the pathways of flavonoid biosynthesis in model and crop plants. J Exp Bo.

[32] Assenov Y, Ramírez F, Schelhorn SESE, Lengauer T, Albrecht M (2008). Computing topological parameters of biological networks. Bioinformatics.

[33] Wittstock U, Halkier BA (2002). Glucosinolate research in the *Arabidopsis* era. Trends Plant Sci.

[34] Altaf-Ul-Amin M, Katsuragi T, Sato T, Ono N, Kanaya S (2014). An 460 unsupervised approach to predict functional relations between genes based on 461 expression data. BioMed Res Int.

[35] Nakamura Y (2014). KNApSAcK metabolite activity database for retrieving the relationships between metabolites and biological activities. Plant Cell Physiol.

[36] Dinchev D, Janda B, Evstatieva L, Oleszek W, Aslani MR, Kostova I (2008). Distribution of steroidal saponins in *Tribulus*
*terrestris* from different geographical regions. Phytochemistry.

[37] Teles YCF, Souza MSR, De Souza M, Def V (2018). Sulphated flavonoids: biosynthesis, structures, and biological activities. Molecules.

[38] Barron D, Varin L, Ibrahim RK, Harborne JB, Williams CA (1988). Sulphated flavonoids-an update. Phytochemistry.

[39] Barabási AL, Oltvai ZN (2004). Network biology: understanding the cell’s functional organization. Nat Rev Genet.

[40] Kim S, Thiessen PA, Bolton EE, Chen J, Fu G, Gindulyte A, Han L, He J, He S, Shoemaker BA, Wang J, Yu B, Zha J, Bryant SH (2016). PubChem substance and compound databases. Nucleic Acids Res.

[41] Kanehisa M (2016). KEGG bioinformatics resource for plant genomics and metabolomics. Methods Mol Biol.

[42] Kanehisa M, Goto S (2000). KEGG: Kyoto encyclopedia of genes and genomes. Nucleic Acids Res.

[43] Guijas CJ, Montenegro-Burke R, Domingo-Almenara X, Palermo A, Warth B, Hermann G, Koellensperger G, Huan T, Uritboonthai W, Aisporna AE, Wolan DW, Spilker ME, Benton P, Siuzdak G (2018). METLIN: A Technology platform for identifying knowns and unknowns. Anal Chem.

[44] Cao Y, Charisi A, Cheng LC, Jiang T, Girke T (2008). ChemmineR: a compound mining framework for R. Bioinformatics.

[45] Patterson DE, Cramer RD, Ferguson AM, Clark RD, Weinberger LE (1996). Neighborhood behavior: a useful concept for validation of ‘molecular diversity’ descriptors. J Med Chem.

[46] Liu K, Morita AH, Kanaya S, Atlaf-Ul-Amin M (2018). Metabolite-content-guided prediction of medicinal/edible properties in plants for bioprospecting. Curr Res Complement Altern Med.

[47] Martin YC, Kofron JL, Traphagen LM (2002). Do structurally similar molecules have similar biological activity?. J Med Chem.

[48] Wijaya SH, Husnawati H, Afendi FM, Batubara I, Darusman LK, Altaf-Ul-Amin M, Sato T, Ono N, Sugiura T, Kanaya S (2014). Supervised clustering based on DPClusO: Prediction of plant-disease relations using Jamu formulas of KNApSAcK database. Biomed Res Int.

[49] Letunic I, Bork P (2016). Interactive tree of life (iTOL) v3: an online tool for the display and annotation of phylogenetic and other trees. Nucleic Acids Res.

[50] Imanishi T, Nakaoka H (2009). Hyperlink management system and ID converter system: Enabling maintenance-free hyperlinks among major biological databases. Nucleic Acids Res.

[51] Xiao JF, Zhou B, Ressom HW (2012). metabolite identification and quantitation in LC-MS/MS-based metabolomics. Trends Analyt Chem.

